# Evaluation of hand bone loss by digital X-ray radiogrammetry as a complement to clinical and radiographic assessment in early rheumatoid arthritis: results from the SWEFOT trial

**DOI:** 10.1186/1471-2474-14-79

**Published:** 2013-03-05

**Authors:** Hamed Rezaei, Saedis Saevarsdottir, Pierre Geborek, Ingemar F Petersson, Ronald F van Vollenhoven, Kristina Forslind

**Affiliations:** 1 Unit of Clinical Therapy Research, Inflammatory Diseases (ClinTRID), The Karolinska Institute, Stockholm, SE 171 76, Sweden; 2 Department of Rheumatology, The Karolinska Institute and Karolinska University Hospital, Stockholm, Sweden; 3 Department of Rheumatology, Lund University Hospital, Lund, Sweden; 4 Orthopedics and Rheumatology, Clinical Sciences, Lund University, Lund, Sweden; 5 Department of Medicine, Section of Rheumatology, Helsingborg Hospital, Helsingborg, Sweden

## Abstract

**Background:**

To investigate hand bone loss (HBL) measured by digital X-ray radiogrammetry (DXR) in patients with early rheumatoid arthritis (RA) receiving different treatment regimens, and to evaluate if DXR change rates during the first 12 months correlate with radiological damage after 24 months.

**Methods:**

From the total SWEFOT trial population, 159 patients had hand radiographs correctly timed and taken with same modality to be analyzed with DXR. All patients started treatment with methotrexate. After 3–4 months, patients with DAS28 > 3.2 were randomized to add sulfasalazine and hydroxychloroquine (triple therapy) or infliximab (MTX + INF). Those with DAS28 ≤3.2 were followed in regular care. Radiographic progression over 24 months was scored according to the Sharp van der Heijde score (SHS) and defined as >5 increase in T-SHS over 24 months. Hand bone mineral density (BMD) was measured by DXR at inclusion and 12 months and a change ≥2.5 mg/cm^2^/month was used as a cut-off for HBL.

**Results:**

In the MTX responders, triple therapy, and MTX + INF groups, the proportions with HBL were 4.1%, 22.2% and 16.4%, respectively (p = 0.01), and the mean (SD) radiological progression in these groups was 3.91 (6.72), 7.40 (14.63) and 2.72 (4.55) respectively (p = 0.06). Patients with HBL had significantly greater risk for radiographic progression, compared with patients without HBL (odds ratio 3.09, 95% CI =1.20–7.79, p = 0.02).

**Conclusions:**

Non-responders to MTX had a significantly greater risk of HBL than MTX-responders, despite the add-on therapies. Patients with HBL during the 12 months had greater risk of radiographic progression after 24 months. Evaluation of HBL may help to identify patients who are at risk of radiographic progression.

## Background

Chronic synovitis in rheumatoid arthritis (RA) can lead to irreversible joint damage, which is seen on conventional plain radiography [1]. Measurement of the degree of joint damage represents an important tool to assess disease progression and effectiveness of current treatments [2,3]. Periarticular osteopenia, erosions and joint space narrowing are radiographic features of RA that can be seen on conventional radiography of the hands and feet [3,4]. Of these, periarticular osteopenia, reflecting a reduction in bone mineral density (BMD), is one of the earliest manifestations, and may precede erosion and joint space narrowing [4]; it may be caused by local release of inflammatory mediators and immobility [5,6]. The sensitivity of conventional radiography regarding osteopenia is limited, as it can only be detected if the reduction of bone density is more than 35–50% [7,8]. In recent years, studies have been presented on an alternative method for ascertaining inflammation-related osteopenia in patients with RA, measuring BMD in the diaphyses of the 2^nd^, 3^rd^ and 4^th^ metacarpal bone on conventional radiographs of the hands by digital X-ray radiogrammetry (DXR) [5,9-13]. DXR is a computerized version of the earlier technique of radiogrammetry, measuring cortical bone thickness as originally proposed by Barnett and Nordin [14]. Prior studies have suggested that this new technique has predictive value for RA-related joint damages and radiological progression [7,9,12,13,15]. Here, we present data on hand BMD change measured by DXR based on part of the SWEFOT (SWEdish PharmacOTherapy) early RA trial population [16].

The aim of this study was to determine whether hand bone loss (HBL) analysed with DXR correlated with radiographic progression, as measured by van der Heijde modified Sharp score (SHS) [17] in patients with early RA and to compare HBL and radiographic progression in the three treatment groups of this trial.

## Methods

### Patients

This study consisted of 159 of the 487 patients with early RA who participated in the SWEFOT trial and had correctly timed hand radiographs with sufficient quality to be analyzed with DXR. The SWEFOT trial was a collaboration of 15 rheumatology units in Sweden between 2002 and 2008. At inclusion (baseline), all patients started treatment with methotrexate (MTX) at a dose of 10 mg weekly, which was escalated every 2 weeks by 5 mg up to 20 mg weekly as target dose. All patients received folic acid supplements and their liver enzymes and blood count tests were monitored according to the local guidelines, with dose adjustments if needed, as previously described [16].

After 3–4 months, patients with disease activity score based on 28 joints count (DAS28) >3.2 were randomized in two arms; combination of methotrexate and infliximab [MTX + INF] or MTX, sulfasalazine (SSZ) and hydroxychloroquine (HCQ) [triple therapy]. A total of 147 patients reached low disease activity score (DAS28 ≤3.2) [MTX monotherapy] and were not randomized in the trial but were followed up in regular care as previously described [16,18].

The study was approved by regional ethics committees of all participating units [Karolinska Institute 02-211, Örebro 2002/202 500:16, Umeå 2002, Linköping 02-186, Uppsala Ups 02-241, Göteborg Gbg Ö 282-01, M 088-02, Lund: LU 398-01 and Stockholm Central Ethical Review Board (EPN)2005/1361, 2006/248-3]. All patients received oral and written information prior to inclusion, and consented to participate by signing the informed-consent document. The patients were followed for 24 months.

### Clinical assessment and physical functional

The disease activity was measured by DAS28 based on erythrocyte sedimentation rate (ESR) [19]. ESR was replaced by C-reactive protein (CRP) where ESR was missing [20]. Analysis of anti-citrullinated protein antibodies (ACPA) was made with the standard Enzyme-linked Immunosorbent assay (Immunoscan-RA Mark2 ELISA test, Euro-Diagnostica, Malmö, Sweden). Rheumatoid factor (RF) was measured by standard laboratory methods at the participating clinics. The functional disability was evaluated using the Swedish version of the Stanford Health Assessment Questionnaire disability index (HAQ) [21].

### Radiographic assessment and digital X-ray radiogrammetry (DXR)

Radiographs of hands and feet were performed at baseline, after 12 and 24 months. The radiographic damage was assessed in 144 of 159 patients according to the van der Heijde modified Sharp score (SHS) allowing presentation of total score [T-SHS (range 0–448)], erosion score [ES (range 0–280)] and joint space narrowing score [JSNS (range 0–168)] separately [22]. One of two certified readers (KA, KF) who were blinded for the treatment assignment read each set of radiographs in chronological order. The inter-class correlation coefficient between the readers was 0.94 and smallest detectable change (SDC) was 5.8, calculated by the formula described by Bruynesteyn et al. [23]. Radiographic progression was defined as an increase in T-SHS > 5 units after 24 months [24].

Bone mineral density (BMD) of the hands was measured on hand radiographs in the 159 patients using DXR (the online Pronosco X-posure System, SECTRA), a computerized version of the traditional technique of radiogrammetry measuring cortical bone thickness as originally proposed by Barnett and Nordin [14]. With this method, the narrowest part of the second, third and forth metacarpal bones are identified. In each area, the thickness and porosity of bone cortex are analyzed around the centre and mid-shaft of the metacarpal bone [25]. DXR of both hands were analyzed and the mean of DXR-BMD of both hands was used as a value of DXR-BMD for each patient. DXR-BMD values are given in mg/cm^2^ per month.

The hand radiographs were sorted on case number and modality type. Any image that was derived from a different modality type than other images from the same patient was removed [11]. Images with severely improper positioning for DXR-BMD measurements were also removed.

DXR-BMD was measured at baseline and after 12 months. Moderately elevated bone loss was defined as a change in BMD ≥ 0.25 and < 2.5 mg/cm^2^ per month and highly elevated bone loss was defined as a change in BMD ≥ 2.5 mg/cm^2^ per month (30 mg/ cm^2^ per 12 month), defined by the device manufacturer (Sectra, Sweden) [26]. To make the results in this study as usable as possible for clinical interpretation, as well as for the ability to compare to other cohorts, a fixed threshold level for highly elevated hand bone loss (HBL) of the device, that is a DXR-BMD change ≥ 2.5 mg/cm^2^ per month, was used for analysis.

### Statistical analysis

The distribution of the variables is given as the mean with standard deviation (SD). The Chi-squared test and Fisher´s Exact test were used to compare dichotomous variables between groups and the independent Student’s t-test, paired-sample T-test and ANOVA to compare continuous variables between groups (for pairwise comparison, Bonferroni test was used). For non-normally distributed data, and in particular for the radiological scores, the Mann-Whitney test and Kruskal Wallis test were used for the comparison between two and three groups, respectively. Statistical analysis was performed with SPSS 20 software (SPSS, Chicago, IL, USA).

## Results

### Baseline characteristics

In the SWEFOT trial, the 487 patients had a total of 1203 hand radiographs performed at the different time points; of whom 159 patients had radiographs of the hands, taken within the time-frame of the baseline and 12 months visit ,that qualified for DXR analysis. The main reason for excluding radiographs was that different radiographic modalities had been used at baseline and after 12 months in the same patient. No patient was removed due to severe joint damage or prosthesis since this was an early RA population.These 159 patients did not differ from the whole study population in baseline characteristics, see Table 1. The number of patients (%) in the MTX responder, triple therapy and combination MTX + INF groups was 49 (30.8%), 55 (34.6%) and 55 (34.6%), respectively. There was no difference in the baseline DAS28 values between the randomized groups (p = 1.0), but the MTX responders had lower DAS28, compared with the other two groups (p < 0.05 for comparison with those groups combined or each at a time). The MTX responders group had also a better functional status (measured by HAQ) than the other two groups (p < 0.05). Patients with triple therapy had higher ESR and CRP at baseline, in comparison with the other groups (p < 0.05). No statistically significant differences were seen, neither in T-SHS nor in ES between the three groups at baseline (T-SHS: p = 0.07, ES: p = 0.52). BMD at baseline did not differ between the groups (p = 0.14).

**Table 1:** Baseline characteristics of the SWEFOT participants for all patients and also within each treatment group

	**All SWEFOT patients (N = 487)**	**All study patients (N = 159)**	**MTX responders (N = 49)**	**Triple therapy (N = 55)**	**Combination MTX + INF (N = 55)**	**p-value**
**Disease duration, months**	6.16 (3.20)	6.08 (3.20)	5.86 (3.12)	6.00 (3.10)	6.35 (3.40)	0.79
**Sex, female**	70.6 (344)	72.3 (115)	63.3 (31)	74.5 (41)	78.2 (43)	0.21
**ACPA pos.% (n)**	63.7 (310)	60.3 (91)	55.6 (25)	62.3 (33)	62.3 (33)	0.74
**RF pos.% (n)**	68.4 (333)	69.1 (96)	68.2 (30)	71.4 (35)	67.4 (31)	0.90
**DAS 28**	5.73 (1.01)	5.70 (1.00)	5.23 (0.99)	5.99 (1.02)	5.85 (0.92)	<0.001*/0.004**/1.0^
**ESR (mm)**	39.8 (28.1)	38.6 (26.6)	31.2 (24.4)	49.3 (27.7)	34.4 (24.3)	0.001*/1.0**/0.008^
**CRP (mg/L)**	33.7 (42.4)	35.1 (40.4)	27.3 (40.1)	47.1 (41.2)	29.8 (37.7)	0.04*/1.0**/0.07^
**HAQ**	1.19 (0.58)	1.19 (0.59)	0.97 (0.47)	1.35 (0.65)	1.25 (0.57)	0.003*/0.04**/1.0^
**T-SHS**	4.54 (8.01)	4.79 (7.85)	3.04 (6.00)	5.98 (8.53)	5.08 (8.37)	0.07
**ES**	1.91 (3.75)	1.99 (3.56)	1.71 (2.87)	2.43 (4.32)	1.76 (3.21)	0.52
**DXR-BMD **(g/cm^2^)	----	0.58 (0.08)	0.59 (0.08)	0.57 (0.06)	0.56 (0.08)	0.14

The values are mean (SD) or percentages (%).

*Between MTX-responders and triple therapy.

** Between MTX-responders and combination MTX + INF.

^ Between triple therapy and combination MTX + INF.

Abbreviation: ACPA: Anti-citrullinated protein antibody, RF: Rheumatoid factor, DAS28: 28 joint-based disease activity score, ESR: Erythrocyte sedimentation rate, CRP: C-reactive protein, HAQ: Health assessment questionnaire disability index, T-SHS: Total van der Heijde modified Sharp score, ES: Erosion Score, DXR: Digital X-ray radiogrammetry, BMD: Bone mineral density.

In the entire study group, there was no difference in radiographic progression at 24 months between ACPA positive and ACPA negative patients [change in T-SHS 5.04 (7.42) compared to 4.98 (5.72), p = 0.28]. Neither was any difference observed between RF positive and negative patients [change in T-SHS: 7.33 (16.41) compared to 4.23 (6.33), p = 0.50], or between women and men [change in T-SHS: 5.11 (11.37) vs. 4.03 (5.73), p = 0.65]. Further, patients with radiographic damage at baseline did not have significantly more progression at 24 months, although a trend was observed [5.87 (12.21) vs. 3.32 (5.85), p = 0.15]. Patients with T-SHS > 5 at 12 months had more radiographic progression totally from baseline to 24 months [8.43 (12.93) vs. 0.93 (2.40), p < 0.005 / OR 14.10, 95% CI =5.41–36.73, p < 0.001 (Fisher´s exact test)] and between 12 and 24 months [1.70 (4.13) vs. 0.62 (1.78), p = 0.03 / OR 9.05, 95% CI = 1.99–40.95, p = 0.001 (Fisher´s exact test)]vs. those who had T-SHS ≤ 5 points.

### Three months follow-up visit

At 3 months follow-up, the mean (SD) DAS28 in the MTX responder, triple therapy and MTX + INF groups was 2.41 (0.77), 4.59 (0.96) and 4.86 (1.01), respectively (p < 0.001, between MTX responders and the randomized groups). Overall, MTX responders also had lower ESR, CRP and HAQ, as expected from the trial design. There were no statistically significant differences in ESR and CRP between the triple therapy and MTX + INF groups at 3 months follow-up visit (ESR: p = 0.30; CRP: p = 0.28).

### Clinical characteristics at baseline in patients with and without radiographic progression and HBL

Table 2 shows the baseline characteristics in patients with and without radiographic progression, defined as an increase > 5 units in T-SHS during 24 months, and similarly in patients with and without HBL during the first 12 months. Patients with radiographic progression at 24 months had more inflammatory activity at baseline than patients who did not progress [higher ESR and CRP (p < 0.001)]. For patients with or without HBL, baseline CRP differed significantly (p = 0.004) and a numeric difference was seen for ESR (p = 0.12).

**Table 2:** **Baseline characteristics in the patients with complete clinical and radiographic data divided by: A) radiological progression or not during the first 24 months (increase in total SHS score > 5 points) and B) hand bone loss or not during the first 12 months (DXR-BMD change rate ≥ 2.5 mg/cm**^**2**^**/month)**

	**Radiological progression**			**Hand bone loss**		
	**YES (N = 43)**	**NO (N = 101)**	**p-value**	**YES (N = 23)**	**NO (N = 126)**	**p-value**
**Symptom duration, months**	6.25 (3.27)	5.98 (3.15)	0.70	5.97 (3.13)	6.70 (3.66)	0.30
**T-SHS = 0,%**	27.9	47.5	**0.04**	30.5	43.7	0.26
**ES =0,%**	46.5	63.4	**0.04**	47.8	59.5	0.36
**DAS 28**	5.89 (1.01)	5.60 (1.00)	0.13	5.86 (0.95)	5.66 (0.98)	0.37
**ESR (mm)**	49.8 (30.9)	31.57 (20.9)	**<0.001**	46.6 (25.9)	37.1 (26.7)	0.12
**CRP (mg/L)**	47.7 (41.7)	27.2 (36.2)	**<0.001**	56.9 (47.4)	30.8 (37.6)	**0.004**
**RF pos.%**	38.3	28.6	0.30	35.5	30.5	0.68
**ACPA pos.%**	64.0	41.4	0.60	56.5	61.4	0.65

Abbreviation: T-SHS: Total van der Heijde modified Sharp score, ES: Erosion Score, DAS28: 28 joint-based disease activity score, ESR: Erythrocyte sedimentation rate, CRP: C-reactive protein, RF: Rheumatoid factor, ACPA: Anti-citrullinated protein antibody.

### Radiographic progression measured by the van der heijde modified sharp score (SHS)

The mean (SD) T-SHS at baseline, 12 and 24 months was 4.79 (7.85), 8.58 (12.72) and 9.76 (13.59), respectively (p < 0.001). At the same time points the numbers for ES was 1.91 (3.75), 3.41 (6.10) and 3.70 (6.30), respectively (p < 0.001). The mean (SD) increase in T-SHS at 12 and 24 months in the whole group was 3.69 (8.63) and 4.81 (10.11), respectively. There was a numerical but statistically non-significant difference in radiographic progression over 24 months between the MTX monotherapy [3.91 (6.72)] and triple therapy [7.40 (14.63)] groups. Patients with triple therapy tended to have more radiographic progression than patients receiving MTX + INF [2.72 (4.55)] (p = 0.06). A similar analysis for increase in ES at 24 months showed a mean (SD) of 1.82 (4.23), 2.42 (6.31), and 0.53 (3.03) in patients with MTX monotherapy, triple therapy, and MTX + INF (p = 0.06, between groups) respectively.

### Hand bone loss (HBL)

**Figure 1:**
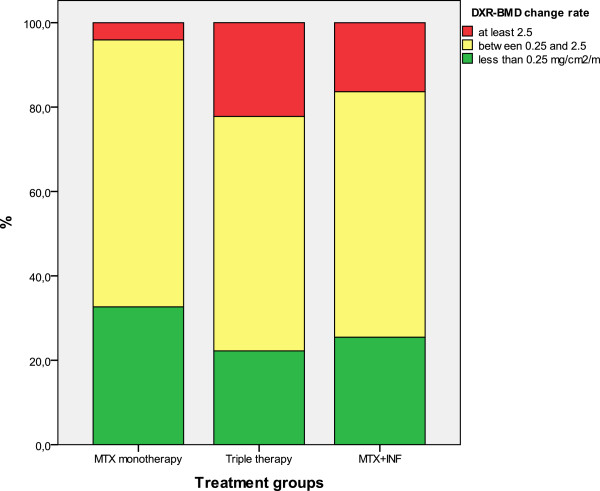
**The proportion of patients with different amount of hand bone loss (HBL) during the first 12 months in each treatment group. **Only 4.1% of patients with MTX monotherapy had HBL during the first 12 months (p = 0.01, between MTX monotherapy and randomized groups). The number of patients with HBL in each therapy group: MTX responder: 2/49; Triple therapy: 12/55; MTX + INF: 9/55.

DXR was analyzed in 159 patients and HBL was found in 23 (MTX monotherapy 2, triple therapy 12 and MTX-INF 9). The mean (SD) DXR-BMD change in all patients was −1.06 (1.44) mg/cm^2^/month, and was non-significantly lower in the MTX monotherapy group than in the triple therapy and MTX + INF groups [−0.65 (1.02), −1.22 (1.54) and −1.26 (1.59) mg/cm^2^/month, respectively; p = 0.08 between MTX monotherapy and the randomized groups]. The proportion of patients who had DXR-BMD change above median (0.71 mg/cm^2^/month) was 40.8% in the MTX monotherapy group, 50.0% in triple therapy and 58.2% in the MTX + INF treated group (p = 0.2). Figure 1 shows the proportion of patients with normal, elevated and highly elevated DXR-BMD change in each treatment group. Only 4.1% of patients in the MTX monotherapy group had HBL during the first 12 months, while this was observed in 22.2% and 16.4% of the patients receiving triple therapy and MTX + INF, respectively (p = 0.01, for MTX monotherapy vs. randomized groups).

### HBL and radiographic progression

**Figure 2:**
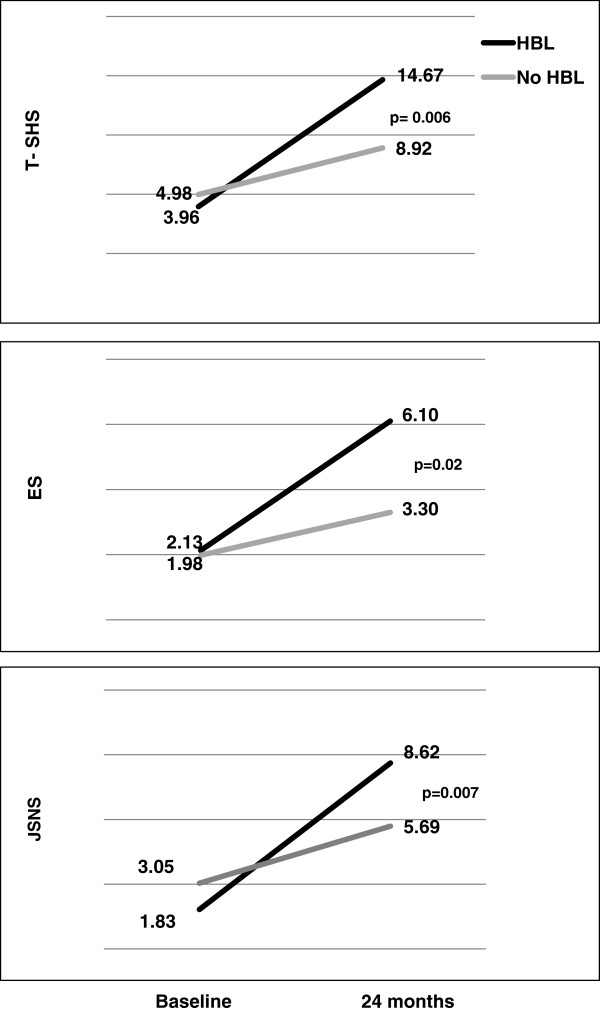
Radiographic progression according to the three parameters of SHS (T-SHS: Total van der Heijde modified Sharp score, ES: Erosion Score and JSNS: joint space narrowing score) during the first 24 months in patients with (black lines) and without (gray lines) hand bone loss (HBL) during the first 12 months of follow-up.

**Figure 3:**
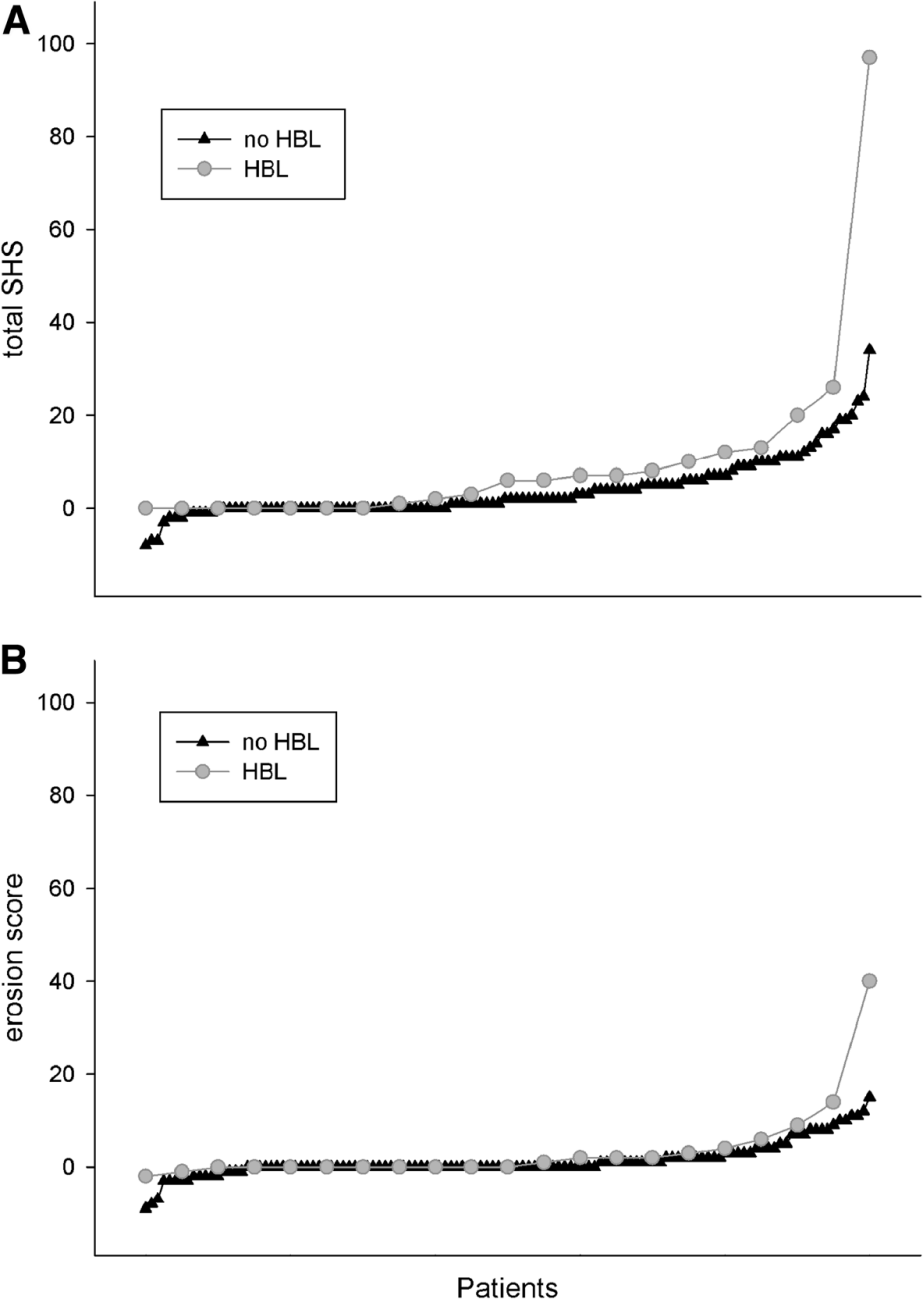
**Radiographic progression (A: total van der Heijde modified Sharp score and B: erosion score) during 24 months in patients with (n=23) and without (n=121) hand bone loss (HBL). **The probalility plot depicts individual radiographic progression for each patient in ascending order. Radiographic progression is none or minimal for most patients (median: 0), but with a difference in progression between the groups at the higher end.

Patients with HBL had significantly more radiographic progression over 24 months [10.38 (21.08)] than the patients without HBL [3.86 (6.42)] as shown in Figure 2 (p = 0.006). Analysis of the ES and JSNS (Figure 2) showed similar patterns. Radiographic progression between 12 and 24 months was also statistically significant in the patients with HBL compared to those without [2.90 (3.64) vs. 0.88 (3.12) (p = 0.005)]. Figure 3 illustrates also well that most of the patients do not have radiographic progression (30/44 in the monotherapy, 30/53 in triple therapy and 34/47 in MTX + INF group, respectively). DXR (cut-off > 2.5 mg/cm2/month) had 89% specificity to predict radiographic progression (T-SHS > 5) but the sensitivity was only 26%.

In the triple therapy group, the mean (SD) increase in T-SHS was 15.42 (26.65) in patients with, and 5.10 (7.73) in patients without HBL (p = 0.03). The mean (SD) increase in ES was 5.42 (11.23) and 1.50 (3.73) in patients with HBL vs. those without HBL, respectively (p = 0.06). Such differences were not seen in the MTX monotherapy and MTX + INF treated groups (data not shown).

Patients with HBL had significantly greater risk of radiographic progression (>5 increase in T-SHS) over 24 months (odds ratio 3.09, 95% CI =1.20–7.79, p = 0.02). This was most marked and only statistically significant in the group of patients receiving triple therapy (odds ratio 4.15, 95% CI = 1.05–16–35, p = 0.04), but not in the MTX monotherapy group (odds ratio 2.50, 95% CI = 0.14–43.28, p = 0.50) or the MTX + INF group (odds ratio 1.88, 95% CI = 0.30–11.77, p = 0.50), particularly due to a limited number of patients who had HBL (n = 2 and 9 in MTX monotherapy and MTX + INF, respectively).

### Treatment changes in patients with and without radiographic progression or HBL

During the first 12 months of the trial, 19.4% of the patients changed treatment strategies because of lack of treatment efficacy or drug side effects. In the triple therapy group, 32.7% of patients changed treatment over 24 months (most of them changed to treatment with TNF inhibitors, data not shown). These patients had more HBL than those who continued their treatment according to the protocol [−2.05 vs. −0.81 mg/cm^2^/month (p = 0.004)], but there was no difference in radiographic progression between these two groups (p = 0.95). Eight patients on combination therapy with MTX + INF discontinued treatment during the first 12 months, and their radiographic progression or proportion with HBL did not differ from those who continued their treatment according to protocol.

## Discussion

In this trial based study we present 24 months follow-up of 159 early RA patients in three different treatment groups who had radiographic data of the hands correctly timed and with sufficient quality to be analyzed with DXR. We show that HBL over the first 12 months, measured by DXR is a predictor for radiographic progression at 24 months as well as between 12 and 24 months. This is in agreement with previous studies [9,10,13,27]. Less HBL was observed in patients who had a good clinical response to MTX after 3 months. Patients who were randomized to triple therapy had more often HBL than MTX + INF group, and they also had a greater risk for radiographic progression if they had HBL. Thus, our findings confirm and extend previous findings on the predictive value of HBL measurement for radiographic progression. Stewart et al. have previously shown in an observational study that measurement of HBL, using DXR, at 12 months correlated with erosive changes in patients with early RA and predicted radiographic progression at 48 months follow-up [27].

Hoff et al. also showed that patients with HBL at 12 months had more radiographic damage at 5 and 10 years in comparison with patients without HBL. In line with our results, mean SHS change was 3.6 and 7.1 after 12 and 24 months, respectively. Patients with HBL had higher risk for radiographic damage after 5 and 10 years with an odds ratio (95% CI) of 3.5 (1.42–8.75) and 3.5 (1.43–8.35), respectively. In our study, the corresponding value is 3.09 (1.20–7.79) after 24 months. In Hoff´s study, the least significant change (LSC) was calculated and used as cut-off to define HBL [11].

In the study by Forslind et al. in patients from the BARFOT study, HBL was defined as a change in DXR-BMD by more than 0.0048 g/cm^2^ (4.8 mg/cm^2^/12 month), the smallest detectable change (SDC), during the first 12 month. Significant correlation was observed between HBL during the first 12 months and T-SHS, ES and JSNS during 24 months. Patients with HBL also had a greater risk for radiographic progression with an odds ratio (95% CI) of 3.0 (1.3–7.4) [9] which is in line with this study. In another study from “the Lund early RA cohort”, the same cut-off (4.8 mg/cm^2^/12 month) was used. In that study, it was demonstrated that HBL at 12 months was associated with an elevated Larsen score at year 10 [12].

The main difference between the present and prior studies is that the fixed threshold levels, recommended by the device manufacturer (Sectra, Sweden), were used for analysis, to make the findings as usable as possible for clinical interpretations as well as for the ability to compare it with other cohorts. DXR-BMD change rates were grouped into normal, elevated and highly elevated bone loss, with threshold levels of 0.25 mg/cm^2^/month and 2.5 mg/cm^2^/month [26] and HBL was defined as DXR-BMD change rate ≥ 2.5 mg/cm^2^/month. This value is higher than the thresholds used in previous studies. Nevertheless, the findings are similar using different cut-offs in different studies, indicating that the correlation between HBL and radiologic progression is not only dependent on the particular threshold used in each study cohort.

Good responders to MTX after 3 months were not randomized in the SWEFOT trial and just continued MTX as monotherapy. This group had less HBL, but radiographic damage was more pronounced compared to the patients who received combination MTX + INF. In the BeSt study, patients with initial monotherapy had significantly more HBL than patients on the initial combination therapy [24]. In the PREMIER study, HBL was also less pronounced in patients with combination therapy and significant differences in HBL and radiographic progression were seen between combination therapy (adalimumab + MTX) and MTX monotherapy at 12 and 24 months follow-up [28]. The difference between this study and those studies is that our patients who had good clinical response to MTX monotherapy had less HBL, and a plausible explanation is that our patients had already demonstrated good clinical response to MTX as monotherapy whereas in BeSt and PREMIER, patients were randomized from baseline. However, in this study, patients in the MTX monotherapy group also had more radiographic progression according to T-SHS than patients with combination MTX + INF, confirming the previously shown positive effect of anti-TNF therapy on radiographic progression [28].

One limitation of this study was that the radiographs were not taken for the aim of DXR analysis from the beginning of the trial. As a consequence, for some patients, the baseline and 12-month radiographs were not taken using the same type of instrument and these images could not be analyzed, but if that would not have been the case, few images would have had to be excluded from the study. Thus, if the DXR technology is considered in advance and the images taken consistently with the same equipment, there should not be a significant limitation for DXR in practice. The manufacturer reports a failure rate below 1/100. Another limitation of our study was the relatively small number of patients (23 patients with HBL at 12 months) which limited the power of the study to detected small differences and associations. This small number of patients also resulted in low sensitivity.

Another disadvantage of the current study design was that the assessment of HBL by DXR was done after 12 months. According to current recommendations, treatments of early RA should be evaluated earlier [29]. Therefore, future studies should preferably investigate whether DXR after 3 or 6 months could provide useful information.

However we agree with Forslind et al. [13] that DXR may have a role in predicting destructive rheumatoid arthritis in clinical praxis where/when the radiographs cannot be analyzed according to the scoring systems used in trials.

## Conclusion

In summary, DXR provides information on HBL, observed in patients with early RA. Non-responders to MTX had significantly greater risk for HBL than MTX-responders despite the add-on therapies, and patients with HBL had significantly greater radiographic damage after 24 months. Thus, information from DXR may be complementary to that obtained by clinical assessments and standard radiography.

## Competing interests

KF has received honoraria from Abbott and Bristol-Myers Squibb, not related to this study.

RvV has received research funding and/or honoraria from Abbott, Bristol-Myers Squibb, GSK, Merck, Pfizer, Roche, and UCB Pharma.

IP has received research funding and/or honoraria from Abbott and Pfizer, not related to this study.

The other authors have no competing interests.

## Authors’ contributions

All authors have collaborated in writing and review of this manuscript.

## Pre-publication history

The pre-publication history for this paper can be accessed here:

http://www.biomedcentral.com/1471-2474/14/79/prepub
